# Simultaneous Determination of Four DNA bases at Graphene Oxide/Multi-Walled Carbon Nanotube Nanocomposite-Modified Electrode

**DOI:** 10.3390/mi11030294

**Published:** 2020-03-11

**Authors:** Shuting Wang, Celia Ferrag, Meissam Noroozifar, Kagan Kerman

**Affiliations:** 1 Department of Physical and Environmental Sciences, University of Toronto Scarborough, 1265 Military Trail, Toronto, ON M1C 1A4, Canada; shuting.wang@mail.utoronto.ca (S.W.); celia.ferrag@mail.utoronto.ca (C.F.); m.noroozifar@utoronto.ca (M.N.); 2 Department of Chemistry, University of Toronto, 80 St. George Street, Toronto, ON M5S 3H6, Canada

**Keywords:** graphene oxide, multi-walled carbon nanotube, nanocomposite, guanine, adenine, cytosine, thymine, uric acid, voltammetry

## Abstract

In this study, we developed a modified glassy carbon electrode (GCE) with graphene oxide, multi-walled carbon nanotube hybrid nanocomposite in chitosan (GCE/GO-MWCNT-CHT) to achieve simultaneous detection of four nucleobases (i.e., guanine (G), adenine (A), thymine (T) and cytosine (C)) along with uric acid (UA) as an internal standard. The nanocomposite was characterized using TEM and FT-IR. The linearity ranges were up to 151.0, 78.0, 79.5, 227.5, and 162.5 µM with a detection limit of 0.15, 0.12, 0.44, 4.02, 4.0, and 3.30 µM for UA, G, A, T, and C, respectively. Compared to a bare GCE, the nanocomposite-modified GCE demonstrated a large enhancement (~36.6%) of the electrochemical active surface area. Through chronoamperometric studies, the diffusion coefficients (*D*), standard catalytic rate constant (K_s_), and heterogenous rate constant (K_h_) were calculated for the analytes. Moreover, the nanocomposite-modified electrode was used for simultaneous detection in human serum, human saliva, and artificial saliva samples with recovery values ranging from 95% to 105%.

## 1. Introduction

Deoxyribonucleic acid (DNA) is an important bio-macromolecule which plays a critical role in the storage of genetic information and the overall functioning of biological systems [1,2,3,4,5]. Deoxyribonucleic acid is composed of four nucleobases: guanine (G), adenine (A), thymine (T), and cytosine (C). Both G and A are classified as purines, whereas T and C are known as pyrimidines. These bases are the essential building blocks of the three-dimensional DNA double helix structure, where G is the complementary base with C, and A is the complementary base with T [1,2,3,4,5,6]. The order sequence of these bases in DNA is imperative, as it initiates several meticulous processes such synthesis of proteins by replication and transcription of the genetic information and duplication of the cell [1,4,7].

The abnormal concentration levels and methylation of nucleobases may be associated to DNA damage and diseases such as cancers, HIV/AIDS, myocardial cellular energy status, prostatitis, and Parkinson’s disease [6,8,9,10]. Thus, the multiplexed and sensitive detection of all nucleobases can be of great significance for clinical diagnosis and biomedical applications.

Various analytical techniques have been successfully used for the detection of nucleobases such as capillary electrophoresis (CE) [11], high-performance liquid chromatography (HPLC) [12], ultra-high-performance liquid chromatography combined with electrospray ionization and tandem spectroscopy (UHPLC-ESI-MS/MS) [13], gas chromatography [14], chemical luminescence method [15], spectrophotometric method [16], and Raman spectroscopy [17]. Although these methods are well established and very powerful, they also exhibit some drawbacks such as high-cost, time-consuming procedures, complex operation, and pre-treatment of samples [1,6,18]. To overcome some of these challenges, researchers have been leaning towards developing electrochemical sensors for the analysis of DNA bases. Electrochemical methods display many advantages such as low-cost, simple operation, rapid and real-time detection, high sensitivity, and ease of miniaturization into a portable device [1,6,18].

Nevertheless, electrochemical detection of DNA bases remains challenging because purines and pyrimidine compounds tend to strongly adsorb on the surfaces and high energy is required to oxidize T and C bases. Previously, T and C were assumed to exhibit no electrochemical activity at carbon-based electrodes because of their slow electron transfer kinetics. For example, Xiao et al. [8] fabricated a modified glassy carbon electrode (GCE) using ionic liquid, multi-walled carbon nanotubes (MWCNTs), and gold nanoparticles but could only achieve detection of guanine and adenine. Hason et al. [19] developed a mechanically roughened edge-plan pyrolytic graphite electrode and were still only able to detect the purine nucleobases. Similar attempt was made by Geng et al. [20], where they used a complex three-dimensional WS_2_ nanosheet/graphite microfiber hybrid electrode, but detection for pyrimidine nucleobases was still unachievable. Brett et al. [7] were the first to report the electrochemical oxidation of all four DNA bases at a GCE in highly basic conditions. In their method, linear ranges for detecting all nucleobases were quite narrow: from 0.2 to 10 μM for purines and 1 to 20 μM for pyrimidines [7]. Hence, there is a need to develop a sensor with improved linear ranges and a higher sensitivity for the simultaneous detection of the four nucleobases.

Since uric acid (UA) is the final product formed in the blood as a result of metabolism of purines such as G and A, it is important for the sensor to have the capacity to detect these bases along with UA as an internal standard [21]. The chemical structure of UA is also extremely similar to the structures of G and A purines, thus simultaneously detecting this compound along with the four DNA bases is important to ensure that no interference or overlapping of signals would be observed.

In this work, we developed a novel nanocomposite of graphene oxide (GO)-multi-walled carbon nanotube (MWCNT) hybrid in chitosan (CHT) for the modification of a GCE surface (GCE/GO-MWCNT-CHT) and utilized this modified GCE for the simultaneous determination of all four DNA bases in presence of UA as the internal standard. To the best of our knowledge, no study has been done to detect all four bases simultaneously in the presence of UA using nanocomposite modification of GCE. Both GO and MWCNT have been widely used nanomaterials for the modification of electrodes since they possess excellent mechanical flexibility, bio-compatibility, stability, large surface area, and high electrochemical conductivity [10,22,23,24]. Recently, a novel class of hybrid materials has been introduced which are generated by hybridizing MWCNT with graphene or graphene oxide sheets [25]. These hybrids showed to have better performances in electroanalytical applications compared to when the materials making up the hybrid were used separately. Moreover, this hybrid is beneficial for sensor fabrication because the π–π stacking interaction between the hydrophobic region of GO and the walls of MWCNT assist in avoiding the restacking of the MWCNT and increase the stability of the GO-MWCNT dispersion [25,26]. Additionally, the natural polymer, CHT, is used as a dispersing material in the modification steps of GCE surfaces [22]. An ideal candidate for sensor fabrication is CHT due to its unique properties such high stability, biocompatibility, good adhesion and insolubility in water [27,28]. 

In this study, a set of calibration curves were constructed for the four nucleobases along with uric acid using differential pulse voltammetry (DPV). Scan rate dependence and chronoamperometric studies were also conducted for the determination of effective area, diffusion coefficient (*D*), catalytic standard rate constant (K_s_) and heterogenous electron transfer rate constant (K_h_). Interference, reproducibility and stability studies were also performed using DPV. Most importantly, the nanocomposite-modified GCE was successfully tested for simultaneous determination of G, A, T, C, and UA in real samples such as human blood serum and artificial and human saliva. All experimental data demonstrated that GCE/GO-MWCNT-CHT can serve as an excellent platform for simultaneously detecting all four DNA bases and UA with good sensitivity.

## 2. Materials and Methods

### 2.1. Materials and Reagents 

Uric acid (UA), guanine (G), adenine (A), thymine (T), cytosine (C), graphene oxide (GO, 2 mg/mL, dispersion in water), chitosan (CHI), potassium ferricyanide (III), potassium ferrocyanide (II), potassium chloride, phenol: chloroform: isoamyl alcohol (24:25:1 v/v), acetic acid, hydrochloric acid, sulfuric acid, and human serum (H4522-20 mL) were all acquired from Sigma–Aldrich (Oakville, ON, Canada). Electrolyte solutions of 0.2 M phosphoric acid (Fischer Scientific, Mississauga, ON) were prepared in pH range 2.0 to 8.0 using concentrated NaOH solution for pH adjustment. Multi-walled carbon nanotubes (MWCNT, 13–18 nm) were purchased from Cheap Tubes Inc. (Cambridgeport, VT). Solutions of 1% and 1.5% (w/v) chitosan in 1% (v/v) acetic acid were prepared for electrode modification procedures. Various sizes of 3.0 µm, 1.0 µm, and 0.05 µm alumina powders were obtained from CH Instrumental Inc. (Austin, TX). A solution of 15% (w/v) zinc sulfate and acetonitrile (50/40, v/v) solution was prepared to remove protein in human serum samples. All chemicals were used without further purification and deionized water was used as for the dilutions of all solutions. 

### 2.2. Instrumentation

To characterize the nanocomposite structure of the modified GCE, a Hitachi H-7500 transmission electron microscopy (TEM, Hitachi Ltd., Japan) and an Alpha Fourier transform infrared spectroscopy (FT-IR, Bruker Corp., Germany) were used. All electrochemical measurements were performed at room temperature using μAutolab PGSTAT 128N (EcoChemie, Utrecht, The Netherlands) potentiostat/galvanostat controlled by NOVA™ 2.1 (EcoChemie, Utrecht, The Netherlands) software. The system is a conventional three- electrode cell system where GCE/GO-MWCNT-CHT was the working electrode, a platinum wire was used as the counter electrode and a saturated silver/silver chloride electrode (sat. Ag/AgCl) was used as the reference electrode. Differential pulse voltammetry (DPV) was conducted in a potential window from 0.1 to 1.6 V at a step potential of 5 mV and a modulation amplitude of 0.025 V and modulation time of 0.05 s with an interval time of 0.5 s All electrochemical runs were repeated at least three times, unless otherwise mentioned. Measurement of pH was achieved by using a VWR SB70P pH meter. The sonication of chemical mixtures was performed by a VWR B2500A-DTH ultra-sonicator.

### 2.3. Synthesis of GO-MWCNT Hybrid in CHT

**Scheme 1:**
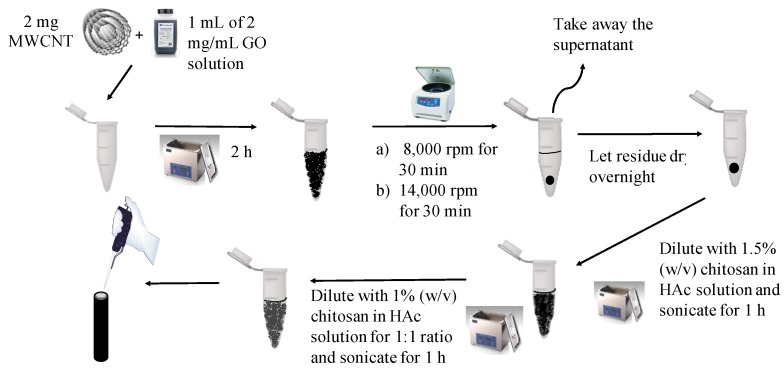
Diagram for the preparation of graphene oxide, multi-walled carbon nanotube (GO-MWCNT) hybrid nanocomposite and the modification of glassy carbon electrode (GCE) surface.

Using a method described by Veerappan et al. [26], GO-MWCNT hybrid was synthesized and dried in open tubes overnight. Then, hybrid nanocomposite (4.1 mg) was mixed with 200 μL of 1.0% (w/v) CHT in 1% acetic acid and was sonicated for 1 h. An aliquot (100 μL) of the mixture was further diluted with 100 μL of 1.5% (w/v) CHT in 1% acetic acid and was sonicated again for 1 h. A flow-chart for preparing GO-MWCNT hybrid nanocomposite in CHT is shown in Scheme 1.

### 2.4. Modification of GCE Surface with GO-MWCNT Hybrid Nanocomposite in CHT

A GCE (3 mm in diameter) was polished mechanically until mirror shiny with alumina powder paste in decreasing sizes from 3.0, 1.0 to 0.05 μm. It was then rinsed with deionized water followed by an ethanol rinse. The CV scanning (15 cycles) were used to activate the surface in 0.5 M H_2_SO_4_ solution with potential ranging from −1.5 V to 1.5 V at scan rate of 100 mV/s. Next, 5 µL of prepared GO-MWCNT hybrid nanocomposite in CHT was drop-cast on the surface of the activated GCE and was left to drying. The nanocomposite-modified GCE was polished electrochemically by running 10 cycles of CV in 0.2 M PBS (pH 7.0) from -0.4 V to 1.6 V at 100 mV/s. 

### 2.5. Spiked Human Serum Sample Preparation (10x dilution)

All experimental studies on human serum samples were performed following the ethical guidelines of the University of Toronto. To prepare the human blood serum samples, the method described by Suh et al. [29] was used to precipitate out the protein in human serum sample. Briefly, 1 mL human serum was mixed in a solution of 0.9 mL 15% (w/v) zinc sulfate and acetonitrile (50/40, v/v). This mixture was then put in ice and vortexed for 20 min. After centrifuging for 5 min at 14,000 rpm for 10 min under room temperature, 0.9 mL supernatant was withdrawn and was diluted with 3.6 mL phosphate buffer solution (0.2 M). Drops of concentrated NaOH solution were added to neutralize the pH of the solution. An aliquot (3 mL) of supernatant was transferred into the cell after another centrifuge cycle. Each standard solution of G (0.0025 M), A (0.0025 M), T (0.005 M) and C (0.005 M) was prepared and gradually spiked into the processed diluted human serum sample. DPV was performed from −1.5 V to +1.5 V at 100 V/s with a step of 0.005 V and an amplitude of 0.025 V. 

### 2.6. Spiked Human Saliva Sample and Artificial Saliva Sample Preparation (5x dilution)

All experimental studies on human saliva samples were performed following the ethical guidelines of the University of Toronto. Saliva samples were collected freshly from a healthy female volunteer and was put into ice bath prior to use. An aliquot (400 μL) of human saliva was then mixed with 1600 μL of phosphate buffer solution (pH 7.0) to serve as electrolyte solution. The same set of four standard nucleobase solutions was then used to spike the diluted human saliva solution. For the preparation of artificial saliva samples, a method described by Madsen et al. [30] was followed. An aliquot (20 mL) of artificial saliva solution (2.5 mM NaHCO_3_, 7.4 mM NaCl, 10 mM KCl, 2 mM CaCl_2_·2H_2_O, 6.4 mM NaH_2_PO_4_·2H_2_O in deionized water) was prepared [30]. Similarly, to the procedure described for human saliva samples, artificial samples were first diluted and then spiked with the standard solutions of four nucleobases for electrochemical measurements.

## 3. Results and Discussion

### 3.1. Characterization of GO-MWCNT Hybrid Nanocomposite in CHT

**Figure 1:**
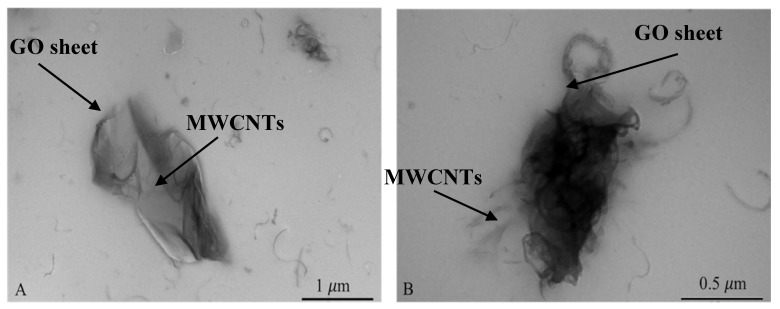
TEM images for the GO-MWCNT nanocomposites in CHT which were used for the modification of GCE surface (scale bars for image (**A**) to (**B**) are 1.0 and 0.5 μm).

Surface morphology of the nanocomposite-modified GCE was investigated using transmission electron microscopy (TEM). As shown in Figure 1A, the hybrid nanocomposite formed a largely entangled complex, which potentially provided lots of active sites for electrochemical reactions. Thin sheets of GO and a large amount of MWCNTs twisting around pores of the complex were observed clearly in Figure 1B. With this rough surface and many highly conductive components, the surface of nanocomposite-modified GCE had the promising potential to promote electrochemical kinetics of analytes with an enhanced electron transfer efficiency. 

**Figure 2:**
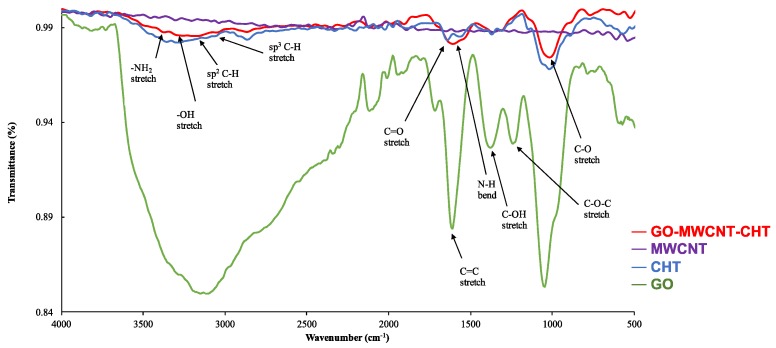
FT-IR spectra for MWCNT, GO, CHT and GO-MWCNT hybrid nanocomposite in CHT.

Nanocomposite structure was also investigated using FT-IR. As shown in Figure 2, FT-IR of CHT had several characteristic peaks for -NH_2_ stretch at ~ 3400 cm^−1^, -OH stretch at ~3200 cm^−1^, sp^3^ C-H stretch at ~2900 cm^−1^, C=O stretch at ~1700 cm^−1^, N-H bend at ~1400 cm^−1^ and C-O stretch at ~1000 cm^−1^. FT-IR spectrum for GO has a very broad and intense peak for -OH stretch at ~3200 cm^−1^, which was expected. Peaks that were attributed to C=O stretch at ~1700 cm^−1^, C=C stretch at ~1600 cm^−1^, C-OH stretch at ~1350 cm^−1^ and C-O-C stretch at ~1200 cm^−1^ were also observed. No obvious transmittance peaks were observed for MWCNTs, which was expected as described in literature [26]. FT-IR spectrum for GO-MWCNT hybrid nanocomposite in CHT had all the characteristic peaks of CHT and GO but with reduced intensity. This result confirmed that hybrid nanocomposite in CHT was successfully synthesized.

### 3.2. pH Effect

**Figure 3:**
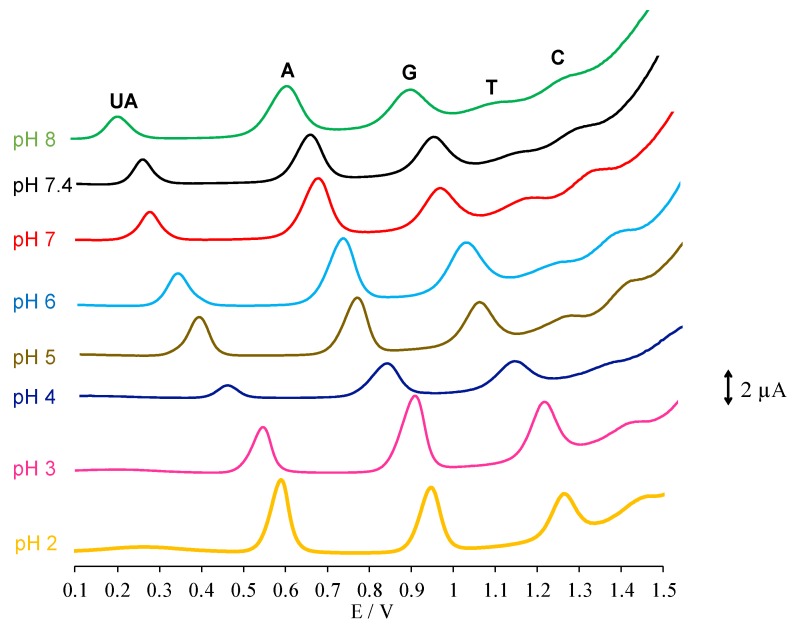
DPV voltammograms using GCE/GO-MWCNT-CHT for pH effect on the simultaneous electrochemical detection of four DNA bases in the presence of UA (pH range: 2.0, 3.0, 4.0, 5.0, 6.0, 7.0, 7.4, and 8.1).

**Figure 4:**
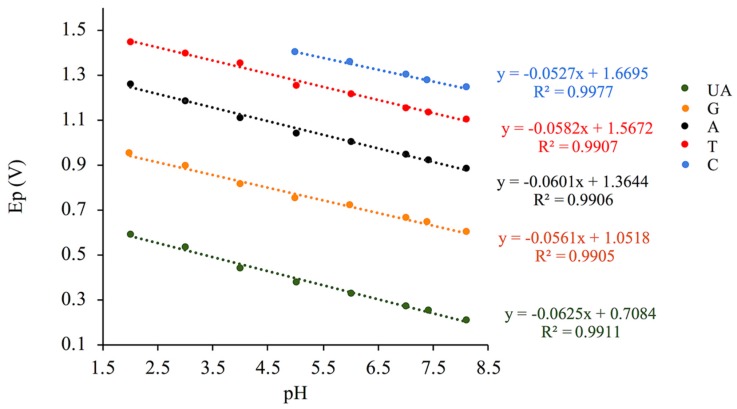
Relationship between pH and E_p_ for the simultaneous electrochemical detection of all four DNA bases in the presence of UA using GCE/GO-MWCNT-CHT.

**Scheme 2:**
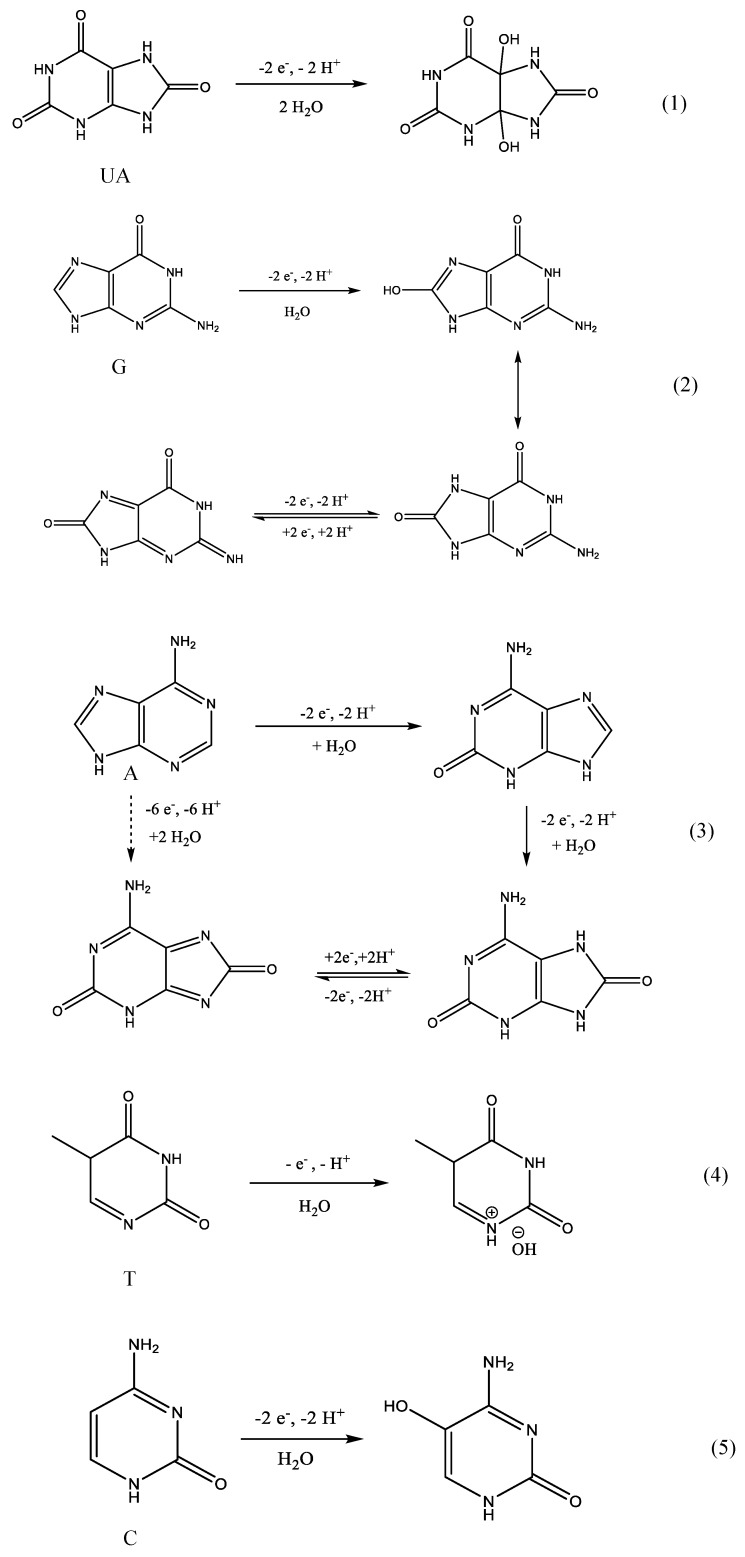
Electrochemical oxidation mechanisms of UA and DNA bases as determined from our experimental data and literature.

Using GCE/GO-MWCNT-CHT, the pH effect of electrochemical detection of the four DNA bases along with UA was investigated using DPV. As depicted in Figure 3, anodic current peaks for five analytes all appeared with pH ranging from 5.0 to 8.0. Indeed, with an increase in pH, analyte peaks shifted towards lower oxidation potentials, which indicated involvement of protons in electrochemical oxidation reactions for each analyte. As shown in Figure 3, under acidic conditions (pH 4.0 and lower), the oxidation peaks for pyrimidine compounds disappeared due to masking of background electrolyte current as a result of oxygen evolution. Optimal pH was determined to be pH 7.0, where an excellent agreement could be reached for relatively low analyte oxidation potentials and high current intensities. All oxidation peaks for the analytes were well distinguished and separated under this physiological pH which also brought convenience for real sample measurements. The linear relationships between pH and oxidation potentials (E_p_) were analyzed as shown in Figure 4. The regression equations extrapolated from Figure 4 are summarized in Table 1. The slopes for plots of E_p_ vs. pH were 62.5, 56.1, 60.1, 58.2, and 52.7 mV/pH for UA, G, A, T and C, respectively. All these values were close to the Nernstian theoretical value of 59.1 mV/pH which indicated that there was an equal number of protons and electrons exchanged for each analyte in the electrochemical oxidation mechanisms [31]. These experimental results matched with previous literature records [11,22,28,31,32,33,34]. As shown in the reaction scheme (1)–(3) and (5) of Scheme 2, two protons and two electrons were postulated to be involved in rate determining steps of electrochemical oxidations of UA G, A, and C [11,22,31,32,34]. While for oxidation of T, only one electron and one proton were involved (reaction scheme (4), Scheme 2) [35]. 

### 3.3. Electrochemical Behavior of UA and Nucleobases 

**Figure 5:**
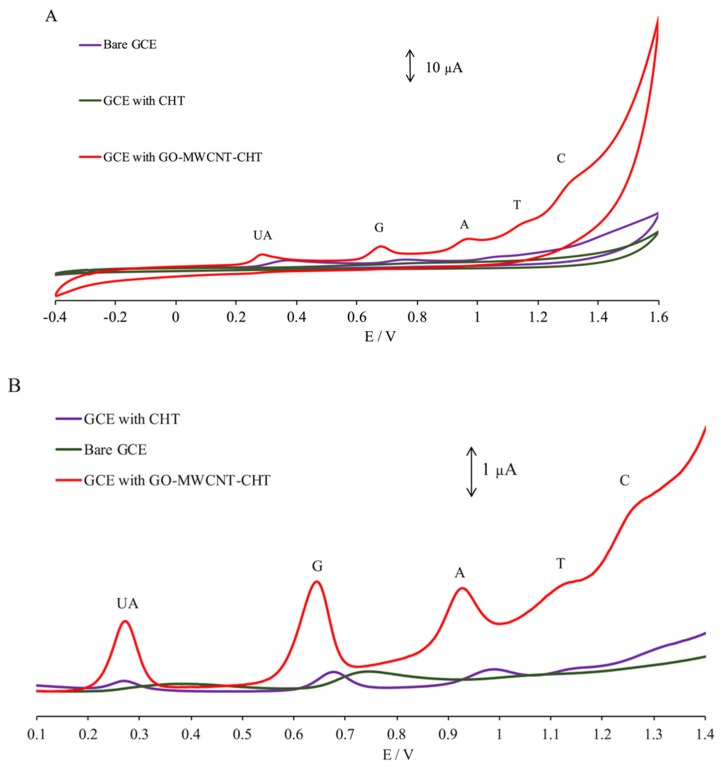
(**A**) Comparison of CV using bare GCE, GCE/CHT, and the GCE/GO-MWCNT-CHT to detect 175 µM UA, 50 µM G, 50 µM A, 250 µM T, and 250 µM C in 0.2 M PBS (pH 7.0); (**B**) comparison of DPV using bare GCE, GCE/CHT, and the GCE/GO-MWCNT-CHT to detect 15 µM UA, 15 µM G, 15 µM A, 35 µM T, and 35 µM C in 0.2 M PBS (pH 7.0).

A comparison study was completed in order to demonstrate that the nanocomposite-modified sensor is able to significantly enhance the electrocatalytic activity. The CV and DPV voltammograms for the simultaneous determination of four DNA bases along with UA as an internal standard using bare GCE, GCE modified with 1% chitosan (GCE/CHT) and the nanocomposite-modified GCE (GCE/GO-MWCNT-CHT) are shown in the Figure 5. In the CV study, there were no significant current peaks observed when using the GCE-CHT, whereas only UA, G, and A were detected using bare GCE with low current peaks at 0.37, 0.77, and 1.07 V, respectively. The GCE/GO-MWCNT-CHT enabled the simultaneous detection of all five analytes with relatively high current peaks. The electrochemical oxidation of all five analytes were irreversible on GCE/GO-MWCNT-CHT as only the anodic peaks were detected at 0.31, 0.69, 0.97, 1.15, and 1.30 V for UA, G, A, T, and C, respectively. Similarly, using DPV, the GCE-GO-MWCNT displayed a more sensitive response to the simultaneous detection of all analytes. The peaks were well-separated and well- distinguishable at 0.28, 0.65, 0.93, 1.13, and 1.27 V for UA, G, A, T, and C, respectively. The DPV of GCE-CHT demonstrated two broad peaks with significantly low current peaks which could not be identified. Based on these CV and DPV results, GCE/GO-MWCNT-CHT was shown to have better overall analytical performance for the simultaneous detection of the four DNA bases along with UA in comparison with control electrodes, bare GCE, and GCE/CHT. 

### 3.4. Simultaneous Electrochemical Detection of Nucleobases in Presence of UA

**Figure 6:**
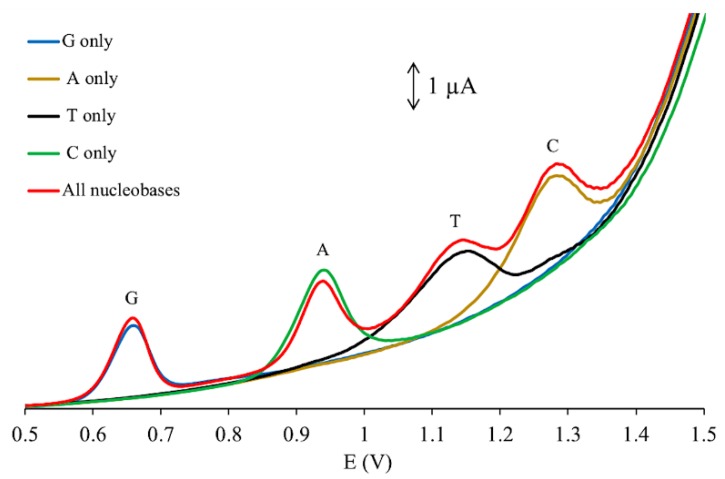
Overlay of DPV measurements using GCE/GO-MWCNT-CHT for individual detections and simultaneous detection of 12.5, 147.5, 97.5, and 10 μM of A, T, C, and G in 0.2 M PBS (pH 7.0).

Individual detections of each four DNA bases were also investigated using DPV. As shown in Figure 6, the peaks were observed around 0.65, 0.93, 1.13, 1.27 V for G (blue), A (brown), T (black), and C (purple), respectively. The overlay in Figure 6 of the individual detections with the simultaneous detection (red) of all four bases demonstrated that there were no significant fluctuations in current response or peak potential when comparing the individual detections (blue, brown, black, and purple lines) with the simultaneous detection (red).

**Figure 7:**
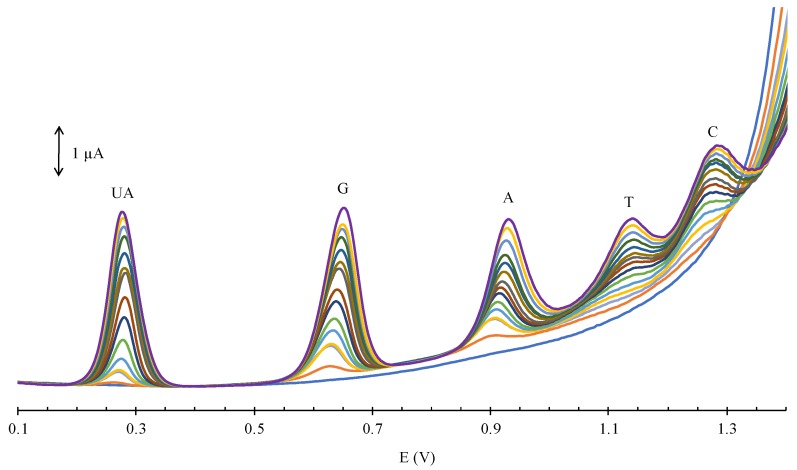
Calibration curves based on DPV for simultaneous detection of UA, G, A, T, and C using GCE/GO-MWCNT-CHT with concentrations ranging from 1.0–151.0, 1.0-78.0, 1.0-79.5, 5.0-267.5, and 12.5–182.5 µM for UA, G, A, T, and C, respectively. The lowest end of the dynamic ranges started from the blue curve and ended in the purple curve for the highest.

**Figure 8:**
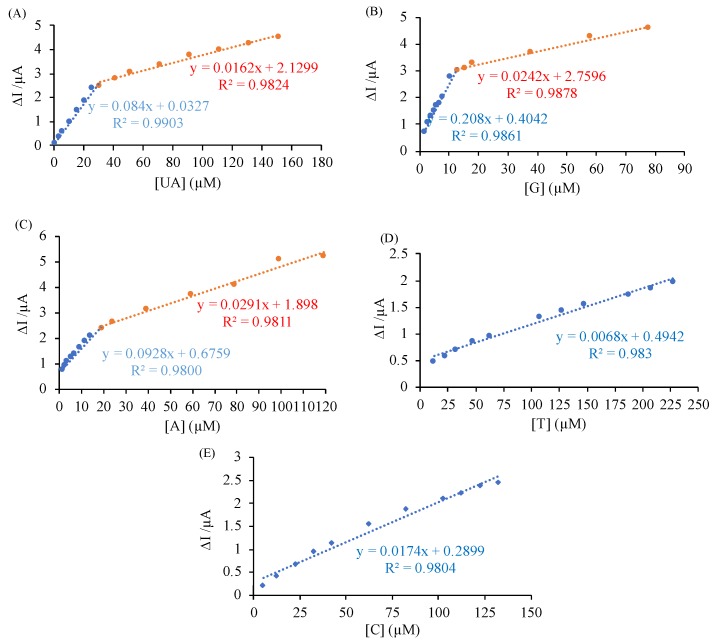
Calibration graphs for UA (**A**) and all four nucleobases (**B**–**E**), respectively.

To build calibration curves, the simultaneous detection of UA and DNA nucleobases using GCE/GO-MWCNT-CHT were investigated using DPV. As all analytes solutions were added into the buffer solution, the intensity of current peaks (I_p_) increased with linear dependence on concentration. Therefore, a set of calibration curves were constructed as shown in Figure 7. Excellent linear dependence was observed between I_p_ and concentrations for each analyte as illustrated in Figure 8. As shown in Figure 8, two linear segments were obtained for UA, G, and A [28,36]. Linear ranges for detecting UA were from 1 µM to 31 µM with calibration Equation (1) and from 31 µM to 151 µM with calibration Equation (2). Similarly, linear responses for G were from 2 µM to 13 µM with calibration Equation (3) and from 13 µM to 78 µM with calibration Equation (4). The linear ranges for A were from 2 µM to 19.5 µM with calibration Equation (5) and from 14.5 µM to 79.5 µM with calibration Equation (6). For T and C, the linear ranges were from 12.5 to 227.5µM and from 5 to 162.5 µM, respectively. Calibration equations for T and C are Equations (7) and (8), respectively. LODs were calculated using 3S_b_/m, where LOD is the detection limit, S_b_ represents the standard deviation of the blank signal, and m represents slope from calibration curves [32,36]. The LODs were determined as 0.12, 0.11, 0.43, 1.71, and 0.80 µM for UA, G, A, T, and C, respectively. In comparison with the other electrochemical ones reported in the literature, this nanocomposite-modified sensor had a promising performance for detecting all four nucleobases in the presence of UA simultaneously, as it demonstrates a low LOD and a wide linear range for each analyte as shown in Table 2 [5,32,33,37,38,39,40,41,42]. The performance between classical chromatography methods and our proposed electrochemical method were compared in Table S2. The proposed modified electrode had the capacity to provide satisfactory recovery results and limit of detection in comparison to classical methods in literature in only 4 min [43,44,45].

ΔI_p1_ (UA) = 0.084 [UA] + 0.0327    (*R*² = 0.9903) (1)

ΔI_p2_ (UA) = 0.0162 [UA] + 2.1299   (*R*² = 0.9828) (2)

ΔI_p1_ (G) = 0.208 [G] + 0.4042      (*R*² = 0.9861) (3)

ΔI_p2_ (G) = 0.0242 [G] + 2.7964      (*R*² = 0.9878) (4)

ΔI_p1_ (A) = 0.0928 [A] + 0.6759      (*R*² = 0.9800) (5)

ΔI_p2_ (A) = 0.0291 [A] + 1.898      (*R*² = 0.9811) (6)

ΔI_p_ (T) = 0.0068 [T] + 0.4826      (*R*² = 0.9849) (7)

ΔI_p_ (C) = 0.0174 [C] + 0.2899      (*R*² = 0.9804) (8)

### 3.5. Interference, Stability, and Reproducibility

A series of interference studies were conducted by increasing the concentration of one analyte while keeping the others at a constant concentration for several measurements (*n* = 10). For instance, as shown in Figure S1I), the current peaks for the electrochemical oxidation of UA increased in a linear fashion with increasing concentrations, while the anodic current for all other four analytes (DNA bases) remained at similar levels without significant variation (± 5%). The same behaviors were observed when varying concentrations of G, A, T, and C as demonstrated in Figure S1. These interference studies are critical when developing a simultaneous sensor platform, because increasing the concentration of one analyte should not have a significant effect on the others. [46] These results demonstrated that there was no significant interference among the analytes and that simultaneous detection of all five analytes was possible using the nanocomposite-modified sensor. Furthermore, reproducibility of our sensor was investigated by evaluating peak current variation of measurements in 0.2 M PBS (pH 7.0) at 30, 57.5, 12.5, 147.5 and 97.5 μM of UA, G, A, T, and C, respectively. The relative standard deviation for successive measurements (n=10) using GCE/GO-MWCNT-CHT were 2.37%, 1.87%, 1.40%, 0.82%, and 0.97% for UA, G, A, T, and C, respectively. These results demonstrated the promising reproducibility of our sensor. Finally, the stability of the sensor was tested for a duration of 14 days. As shown in Figure S2, the results were statistically similar, and the sensor was able to generate repeatable measurements after the 2-week storage period at room temperature. 

### 3.6. Scan Rate Dependence 

**Figure 9:**
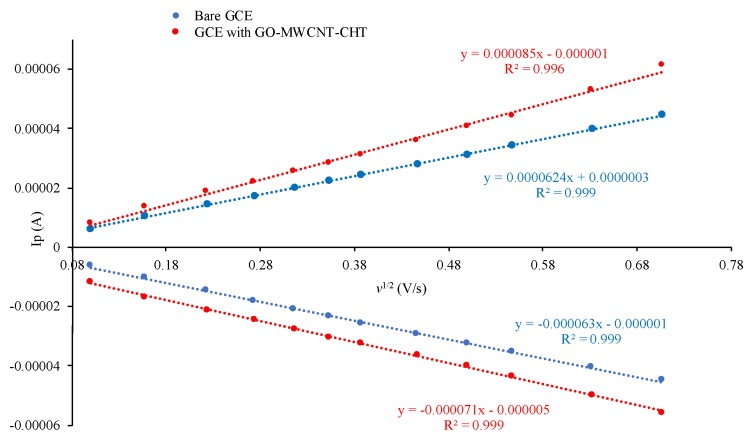
Linear dependence of i_p_ on *v* for bare GCE and GCE/GO-MWCNT-CHT.

The CV was performed at different scan rates to assess the effective surface areas of bare GCE and GCE/GO-MWCNT-CHT in a solution of 1 mM potassium ferrocyanide (K_3_Fe(CN)_6_). The effective areas were calculated using the Randles–Sevcik equation (9) below:

i_p_ = 2.69 ×10^5^ n^3/2^ A C_0_ D ^1/2^*v*^1/2^ (9)

where i_p_ is the measured analyte current, n is the electron transfer number for oxidation of K_3_Fe(CN)_6_ (equal to 1), C_0_ equals to 10^−6^ mol/cm^3^ for the concentration of K_3_Fe(CN)_6_, and D is 7.6 × 10^−6^ cm∙s^−1^ for diffusion coefficient in this process [47]. As shown in Figure 9, both electrodes had excellent linearity between the anodic current and the square root of scan rate. This suggested electrochemical oxidations of all analytes were diffusion-controlled processes using the ferrocyanide probe. Diffusion-controlled processes can be advantageous since they can be a factor to reduce the effect of surface fouling that results from the adsorption of molecules on the electrode surface [8,21]. Slopes of I_p_ vs. v^1/2^ were 0.000062 and 0.000085 A∙s/V for bare GCE and GCE/MWCNT-GO-CHT, respectively. Based on Equation (9), effective areas were calculated as 0.0842 and 0.115 cm^2^ for bare GCE and GCE/MWCNT-GO-CHT, respectively. Therefore, the nanocomposite-modified electrode had a 36.6% larger redox-active surface area which considerably facilitated the electrocatalytic kinetics of analytes. 

### 3.7. Chronoamperometry

**Figure 10:**
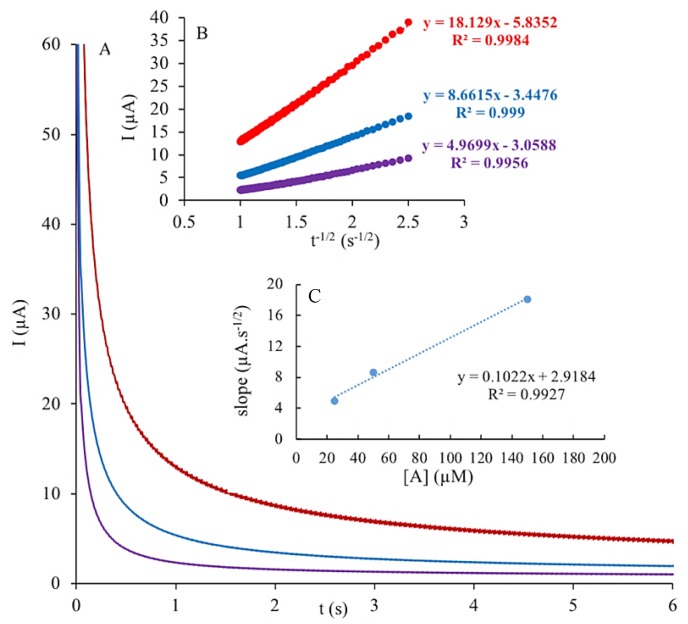
(**A**) Chronoamperograms of GCE/GO-MWCNT-CHT for varying concentrations of adenine (**A**): 25, 50, and 150 μM in 0.2 M PBS (pH 7.0); (**B**) plots of anodic peak currents (I_pa_) versus t^−1/2^; (**C**) plot of the slope of the straight line versus concentration of A.

To determine the diffusion coefficient (*D*) of each analyte as well as the standard kinetic rate constant (K_s_) and heterogeneous kinetic rate constant (K_h_) of electron transfer reactions, chronoamperometric studies were conducted. An example of the chronoamperograms obtained from these studies for analysis of A using GCE/GO-MWCNT-CHT is shown in Figure 10. All other figures of chronoamperometric studies for the detection of UA, G, T and C are displayed in Figures S3–S6, respectively. *D* of each analyte using GCE/ GO-MWCNT-CHT was calculated using Cottrell Equation (10) [36]:

(10) $$i\;=\frac{\mathrm{nFAC}\sqrt{D}}{\sqrt{\pi t}}$$

where i is current in A, n is number of electrons transferred in electrochemical oxidation process, C is the concentration of the analyte, F is Faraday constant (96485 C/mol), *D* is diffusion coefficient, and t is time in s [36].

At GCE/GO-MWCNT-CHT surfaces, *D* was calculated as 1.27 × 10^−6^, 6.91 × 10^−5^, 7.40 × 10^−6^, 4.57 × 10^−4^ and 1.49 × 10^−5^ cm^2^ s^−1^ for UA, G, A, T, and C, respectively. 

In addition, K_h_ was calculated by plotting the i_c_/i_L_ in function of the square root of time (Figures S7–S11). The K_h_ values were calculated by using the slopes obtained from these graph following the equation (11) [47]:

(11) $$\frac{i_{c}}{i_{L\;}}=\;\pi^{\frac{1}{2}}{\left(K_{h}C_{h}t\right)}^{1/2}$$

where i_c_ is the catalytic current, i_L_ is the limiting current with no analyte, C_h_ is the concentration of analyte in electrolyte, and t is time in s [46]. The K_h_ values were determined as 1.45 × 10^3^ M^−1^s^−1^, 3.31 × 10^4^ M^−1^s^−1^, 7.24 × 10^4^ M^−1^s^−1^, 1.39 × 10^2^ M^−1^s^−1^and 1.11× 10^2^ M^−1^s^−1^ for UA, G, A, T, and C, respectively. 

The K_s_ value of each analyte was also determined using the Velasco equation [48]:

(12) $$K_{s}=1.11D_{0}{}^{\frac{1}{2}}{\left(E_{p}-E_{p1/2}\right)}^{-\frac{1}{2}}v^{1/2}$$

where *E*_p_ is oxidation potential, E_p1/2_ is the half-wave oxidation potential, and *D*_0_ is the diffusion coefficient [49]. As shown in Table S1, K_s_ values were determined as 2.00 × 10^−3^, 4.58 × 10^−3^, 1.16 × 10^−3^, 1.12 × 10^−2^, and 2.61 × 10^−3^ cm s^−1^ for UA, G, A, T, and C, respectively. Assuming D_0_ to be 2.00 × 10^−6^ mol^2^ s^−^^1^ for bare GCE, K_s_ values were also calculated as 1.90 × 10^−3^, 1.83 × 10^−3^, 8.46 × 10^−4^, 6.87 × 10^−4^ and 8.26 × 10^−6^ cm s^−1^ for UA, G, A, T and C, respectively. When comparing both bare and modified electrodes, GCE/GO-MWCNT-CHT had a larger K_s_ for G, A, T, and C which indicated more improved electron transfer kinetics at the surface of the nanocomposite-modified GCE. 

### 3.8. Real Samples 

To investigate the application of the nanocomposite-modified sensor in real samples, simultaneous detection of four DNA bases was performed in complex matrices such as saliva and human blood serum. To achieve these measurements the standard addition technique was used. As shown in Figure S12 to S14, standard solutions of G (0.0025 M), A (0.0025 M), T (0.005 M), and C (0.005 M) were used to spike the diluted human serum and saliva samples as well as the artificial saliva ones. Using 0.2 M PBS (pH 7.0), the real samples were diluted 10 fold, 5 fold, and 5 fold for human serum, human saliva, and artificial saliva samples, respectively. Good recovery values were obtained for all samples which ranged from 95% to 105% using the nanocomposite-modified electrode as shown in Table 3. These results demonstrated that our sensor had good potential for applications using real biological fluids.

## 4. Conclusions

In this work, a cost-effective and sensitive nanocomposite-modified sensor was developed for the simultaneous detection of DNA nucleobases along with UA as an internal standard. Compared with other sensors in the literature, it demonstrated wider linear ranges and lower detection limits. Scan rate studies demonstrated that the nanocomposite-modified surface had a large redox-active area that facilitated the electrochemical oxidation of analytes. Important kinetic parameters, such as *D*, K_s_, and K_h_, were calculated based on our chronoamperometric data. Moreover, simultaneous detection was successful in spiking studies using human serum and saliva samples as well as artificial saliva ones with good recovery values. Overall, the sensor had a promising potential to eventually be developed into a diagnostic tool to study DNA damage and DNA methylation in gene expression.

## Figures and Tables

**Table 1:** A summary of the results for the linear dependence of E_p_ on pH for UA and DNA bases using GCE/GO-MWCNT-CHT.

Analytes	Linear Relationships	Slopes (mV/pH)	Regression Coefficients
**UA**	E_p_ (UA) = −0.0625 pH + 0.7084	62.5	*R*² = 0.9911
**G**	E_p_ (G) = −0.0561 pH + 1.0518	56.1	*R*² = 0.9905
**A**	E_p_ (A) = −0.0601 pH + 1.3644	60.1	*R*² = 0.9906
**T**	E_p_ (T) = −0.0582 pH + 1.5672	58.2	*R*² = 0.9907
**C**	E_p_ (C) = −0.0458 pH + 1.6268	52.7	*R*² = 0.9913

**Table 2:** Comparison of the nanocomposite-modified sensor with the ones recorded in the literature for the detection of all four nucleobases.

Electrode	Analytes	Detection Limit (µM)	Linear Range (µM)	Reference
Graphene-Nafion modified GCE	G	0.58	2–120	[37]
	A	0.75	5–170	
MWCNT-Fe_3_O_4_ coated with PDA-Ag modified GPE	G	1.5	8–130	[38]
	A	5.7	0.1–120	
GMC modified GCE	G	0.76	25–150	[39]
	A	0.63	25–200	
PANI-MnO_2_ modified GCE	G	4.8	10–100	[32]
	A	2.9	10–100	
	T	1.3	10–100	
	C	1.3	10–100	
MWCNT-graphite deposited with Au-rGO modified GPE	G	3.3	3–170	[5]
	A	3.7	3–190	
	T	7.9	7.5–800	
	C	9.0	9–900	
Prussian blue/ poly(4-aminosalicylic acid)/ uricase modified graphite screen-printed electrode	UA	3.0	10–200	[40]
Screen-printed graphene electrode	UA	0.4	1.2–1.8	[41]
Poly(DPA) on SiO_2_@Fe_3_O_4_ modified screen-printed graphene electrode	UA	0.20	0.8–2500	[33]
Au-Nafion-modified carbon screen-printed electrode	UA	0.3	0.5–600	[42]
GO-MWCNT hybrid with CHT modified GCE	UA	0.12	1–151	This work
	G	0.11	1–78	
	A	0.43	2–119.5	
	T	1.71	12.5–227.5	
	C	0.80	5–132.5	

PDA: polydopamine; GPE: graphite paste electrode; GMC: graphitized mesoporous carbon; PANI: polyaniline; rGO: reduced graphene oxide; poly(DPA): polydopamine.

**Table 3:** Summary of simultaneous voltammetric detection studies using the spiked human serum, saliva, and artificial saliva samples at GCE/GO-MWCNT-CHT surfaces.

Real Sample	Analytes	Spiked Concentration (μM)	Detected Concentration (μM)	% Recovery
**Human serum**	G	5	5.0	100
	A	10	10.4	104
	T	13.33	13.3	100
	C	83.3	80.8	97
**Human saliva**	G	51.25	52.6	103
	A	32.5	32.4	100
	T	62.5	59.1	95
	C	62.5	64.5	103
**Artificial saliva**	G	50	48.1	96
	A	50	48.2	96
	T	100	98.6	99
	C	100	105.0	105

## Supplementary Materials

The following are available online at https://www.mdpi.com/2072-666X/11/3/294/s1, Figure S1: Differential pulse voltammograms of GCE/MWCNT-GO-CHT for interference studies: I) The concentrations of G, A, T and C were kept constant at 10.5 µM, 12.5 µM, 147.5 µM and 97.5 µM, respectively, while varying the concentrations of UA from 0 to 37.5 µM; II) The concentrations of UA, A, T and C were kept constant at 30 µM, 32.5 µM, 147.5 µM and 97.5 µM, respectively, while varying the concentrations of G from 0 to 28 µM; III) The concentrations of UA, G, T and C were kept constant at 30 µM, 10.5 µM, 147.5 µM and 97.5 µM, respectively, while varying the concentrations of A from 0 to 32.5 µM; VI) The concentrations of UA, G, A and C were kept constant at 30 µM, 10.5 µM, 12.5 µM and 97.5 µM, respectively, while varying the concentrations of T from 0 to 247.5 µM; V) The concentrations of UA, G, A and T were kept constant at 30 µM, 32.5 µM, 147.5 µM and 97.5 µM, respectively, while varying the concentrations of C from 0 to 28 µM. Figure S2: DPV of simultaneous detection of 30, 57.5, 12.5, 147.5 and 97.5 µM of UA, G, A, T and C, respectively, using freshly prepared (0 days) electrode (red line) and stored (14 days at room temperature) electrode (green line). Figure S3: (A) Chronoamperograms of GCE/GO-MWCNT-CHT for varying concentrations of UA: 20, 30, 50 μM in 0.2 M PBS (pH 7.0); (B) Plots of anodic peak currents (I_pa_) vs. t^−1/2^; (C) Plot of the slope of straight line vs. UA concentration. Figure S4: (A) Chronoamperograms of GCE/GO-MWCNT-CHT for varying concentrations of G: 10, 20, 30 μM in 0.2 M PBS (pH 7.0); (B) Plots of anodic peak currents (I_pa_) vs. t^−1/2^; (C) Plot of the slope of straight line vs. G concentration. Figure S5: (A) Chronoamperograms of GCE/GO-MWCNT-CHT for varying concentrations of T: 25, 50, 150 μM in 0.2 M PBS (pH 7.0); (B) Plots of anodic peak currents (I_pa_) vs. t^−1/2^; (C) Plot of the slope of straight line vs. T concentration. Figure S6: (A) Chronoamperograms of GCE/GO-MWCNT-CHT for varying concentrations of C: 25, 100, 125 μM in 0.2 M PBS (pH 7.0); (B) Plots of anodic peak currents (I_pa_) vs. t^−1/2^; (C) Plot of the slope of straight line vs. C concentration. Figure S7: Linear dependence of square root of time on I_c_/ I_L_ for UA detection using chronoamperometry. Figure S8: Linear dependence of square root of time on I_c_/ I_L_ for G detection using chronoamperometry. Figure S9: Linear dependence of square root of time on I_c_/ I_L_ for A detection using chronoamperometry. Figure S10: Linear dependence of square root of time on I_c_/ I_L_for T detection using chronoamperometry. Figure S11: Linear dependence of square root of time on I_c_/ I_L_ for C detection using chronoamperometry. Figure S12: DPV for standard addition in human serum sample that was diluted 10-fold in 0.2 M PBS (pH 7.0). Figure S13: DPV for standard addition in human saliva sample that was diluted 5-fold in 0.2 M PBS (pH 7.0). Figure S14: DPV for standard addition in artificial saliva sample that was diluted 5-fold in 0.2 M PBS (pH 7.0). Table S1: A summary of calculated diffusion coefficients, catalytic rate constants and heterogeneous kinetic rate constant (K_h_) for UA, G, A, T and C using the designated electrode. Table S2: Comparison table of classical methods for DNA detection and the proposed electrochemical method.
