# Supplementary material for: Simultaneous Determination of Four DNA bases at Graphene Oxide/Multi-Walled Carbon Nanotube Nanocomposite-Modified Electrode
Source: Micromachines (Basel). 2020 Mar 11;11(3):294. doi: 10.3390/mi11030294 (PMC7143941; doi:10.3390/mi11030294)
Supplement: Supplementary file 1 [file micromachines-11-00294-s001.pdf]

micromachines

Supplementary Material

Simultaneous Determination of Four DNA bases at Graphene Oxide/Multi-walled Carbon Nanotube Nanocomposite-modified Electrode

Shuting Wang [1,†], Celia Ferrag [1,2,†], Meissam Noroozifar [1] and Kagan Kerman [1,2,*]

[1] Department of Physical and Environmental Sciences, University of Toronto Scarborough, 1265 Military Trail, Toronto, ON M1C 1A4, Canada; shuting.wang@mail.utoronto.ca (S.W.); celia.ferrag@mail.utoronto.ca (C.F.); m.noroozifar@utoronto.ca (M.N.)
[2] Department of Chemistry, University of Toronto, 80 St. George Street, Toronto, ON M5S 3H6, Canada
* Correspondence: kagan.kerman@utoronto.ca
† These authors contributed equally to the work

Table S1. A summary of calculated diffusion coefficients, catalytic rate constants and heterogeneous kinetic rate constant (K_h) for UA, G, A, T and C using the designated electrode.

Analyte	Ep (V)	$Ep_{1/2}$ (V)	D_0 (cm.s^{-1})	Ks (mol^2 s^{-1})	K_h (M^{-1}s^{-1})
UA	0.291	0.252	1.27×10^{-6}	2.00×10^{-3}	1.45×10^{3}
G	0.689	0.284	6.91×10^{-5}	4.58×10^{-3}	3.31×10^{4}
A	0.975	0.298	7.40×10^{-6}	1.16×10^{-3}	7.24×10^{4}
T	1.148	0.701	4.57×10^{-4}	1.12×10^{-2}	1.39×10^{2}
C	1.314	1.045	1.49×10^{-5}	2.61×10^{-3}	1.11×10^{2}

Table S2. Comparison table of classical methods for DNA detection and the proposed electrochemical method.

Method		Linear range (µM)	LOD (µM)	Conditions	Time	Ref
**GC-FID**	A	0.5-50	0.08	Carrier gas: He; initial column temperature set 100 °C for 1 min, followed by rising to 280 °C at 30 °C min−1 up to 280 °C	9 min	[44]
	G	0.5-50	0.09			
	T	0.5-50	0.10			
	C	0.5-50	0.10			
HPLC-DAD	G	0.728-72.79	0.220	mobile phase: acetonitrile (A) and water (B)	50 min	[45]
	T	4.758-475.76	0.260			
HPLC - UV	A	1.11-111.0	0.903	mobile phase: 0.01 mol/L potassium dihydrogen phosphate aqueous solution (A) and methanol (B)	60 min	[46]
	G	0.993-101.3	0.820			
	T	1.19-119.6	0.484			
	C	1.35-136.4	0.270			
Electrochemical Sensor	A	2-119.5	0.430	Electrolyte: Phosphate buffer (pH =7.0), Method: DPV	4 min	This work
	G	1-78	0.110			
	T	12.5-227.5	1.710			
	C	5-132.5	0.800			

44. Brohi, R.O.Z.Z.; Khuhawar, M.Y.; Khuhawar, T.M.J. GC-FID determination of nucleobases guanine, adenine, cytosine, and thymine from DNA by precolumn derivatization with isobutyl chloroformate. J. Anal. Sci. Technol. **2016**, *7*, 0–5.

45. Duan, B.; Wang, L.; Dai, X.; Huang, L.; Yang, M.; Chen, S. Identification and quantitative analysis of nucleosides and nucleobases in aqueous extracts of Fritillaria cirrhosa D. DON. using HPLC-DAD and HPLC-ESI-MS. Anal. Lett. **2011**, *44*, 2491–2502.

46. Cheng, W.; Zhang, X.; Song, Q.; Lu, W.; Wu, T.; Zhang, Q.; Li, C. Determination and comparative analysis of 13 nucleosides and nucleobases in natural fruiting body of Ophiocordyceps sinensis and its substitutes. Mycology **2017**, *8*, 318–326.

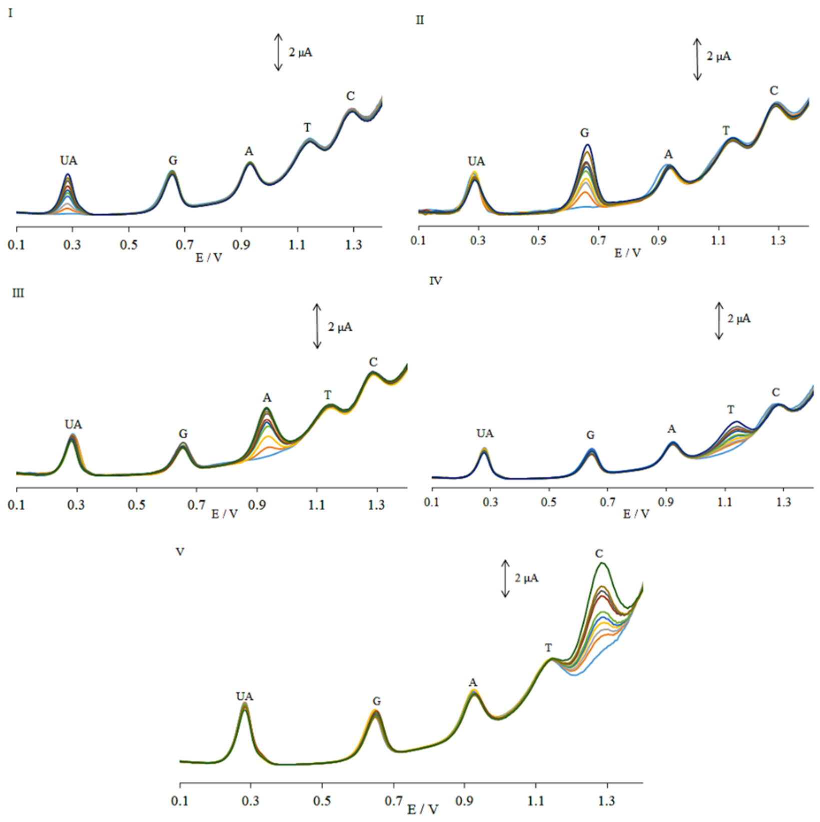

Figure S1. Differential pulse voltammograms of GCE/MWCNT-GO-CHT for interference studies: **I)** The concentrations of G, A, T and C were kept constant at 10.5 μM, 12.5 μM, 147.5 μM and 97.5 μM , respectively, while varying the concentrations of UA from 0 to 37.5 μM; **II)** The concentrations of UA, A, T and C were kept constant at 30 μM, 32.5 μM, 147.5 μM and 97.5 μM , respectively, while varying the concentrations of G from 0 to 28 μM; **III)** The concentrations of UA, G, T and C were kept constant at 30 μM, 10.5 μM, 147.5 μM and 97.5 μM, respectively, while varying the concentrations of A from 0 to 32.5 μM; **VI)** The concentrations of UA, G, A and C were kept constant at 30 μM, 10.5 μM, 12.5 μM and 97.5 μM, respectively, while varying the concentrations of T from 0 to 247.5 μM; **V)** The concentrations of UA, G, A and T were kept constant at 30 μM, 32.5 μM, 147.5 μM and 97.5 μM, respectively, while varying the concentrations of C from 0 to 28 μM.

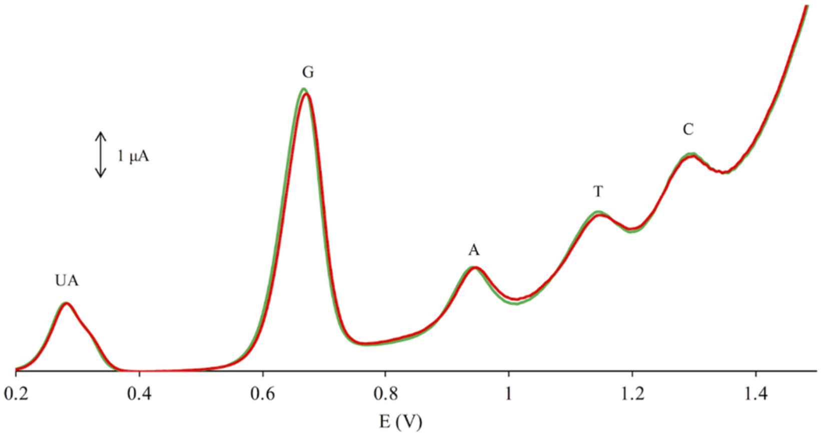

Figure S2. DPV of simultaneous detection of 30, 57.5, 12.5,147.5 and 97.5 μM of UA, G, A, T and C, respectively, using freshly prepared (0 days) electrode (red line) and stored (14 days at room temperature) electrode (green line).

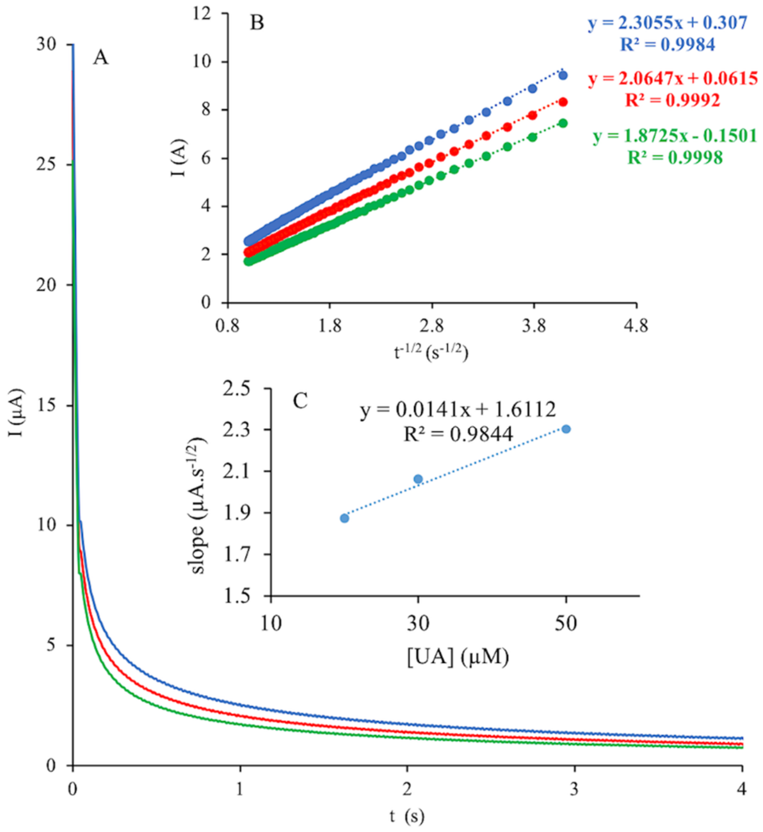

Figure S3. (A) Chronoamperograms of GCE/GO-MWCNT-CHT for varying concentrations of UA: 20, 30, 50 μM in 0.2 M PBS (pH 7.0); (B) Plots of anodic peak currents (I_{pa}) vs. $t^{-1/2}$; (C) Plot of the slope of straight line vs. UA concentration.

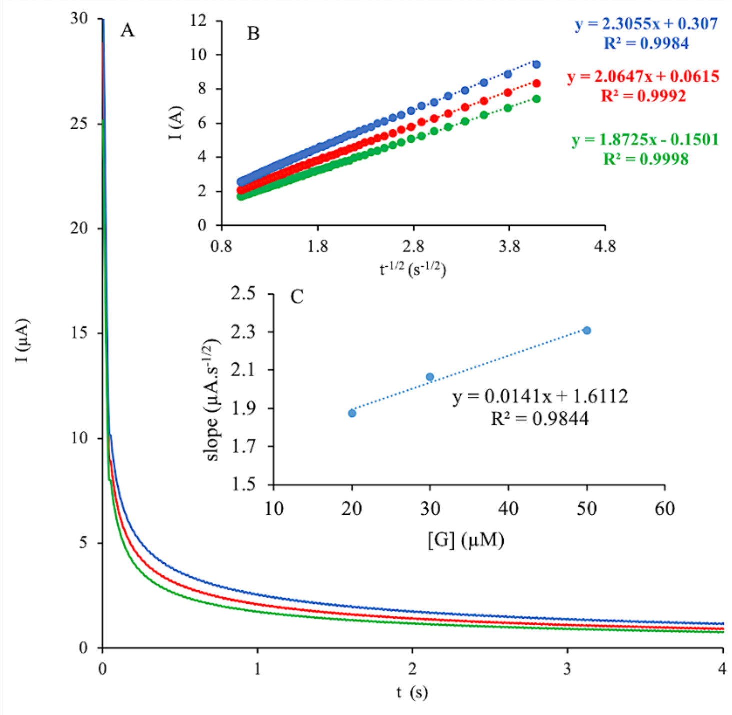

Figure S4. (A) Chronoamperograms of GCE/GO-MWCNT-CHT for varying concentrations of G: 10, 20, 30 μM in 0.2 M PBS (pH 7.0); (B) Plots of anodic peak currents (I_{pa}) vs. $t^{-1/2}$; (C) Plot of the slope of straight line vs. G concentration.

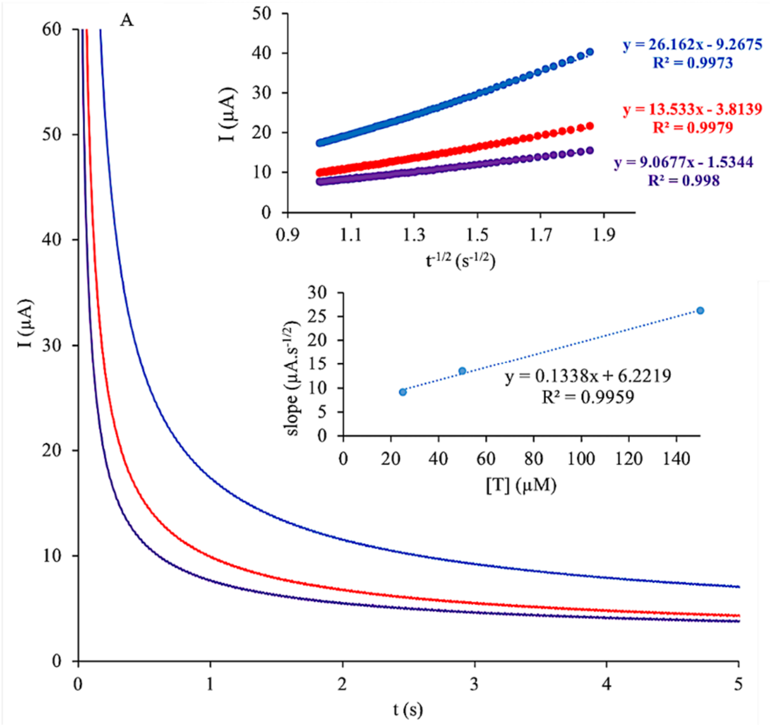

Figure S5. (A) Chronoamperograms of GCE/GO-MWCNT-CHT for varying concentrations of T: 25, 50, 150 µM in 0.2 M PBS (pH 7.0); (B) Plots of anodic peak currents (I_{pa}) vs. $t^{-1/2}$; (C) Plot of the slope of straight line vs. T concentration.

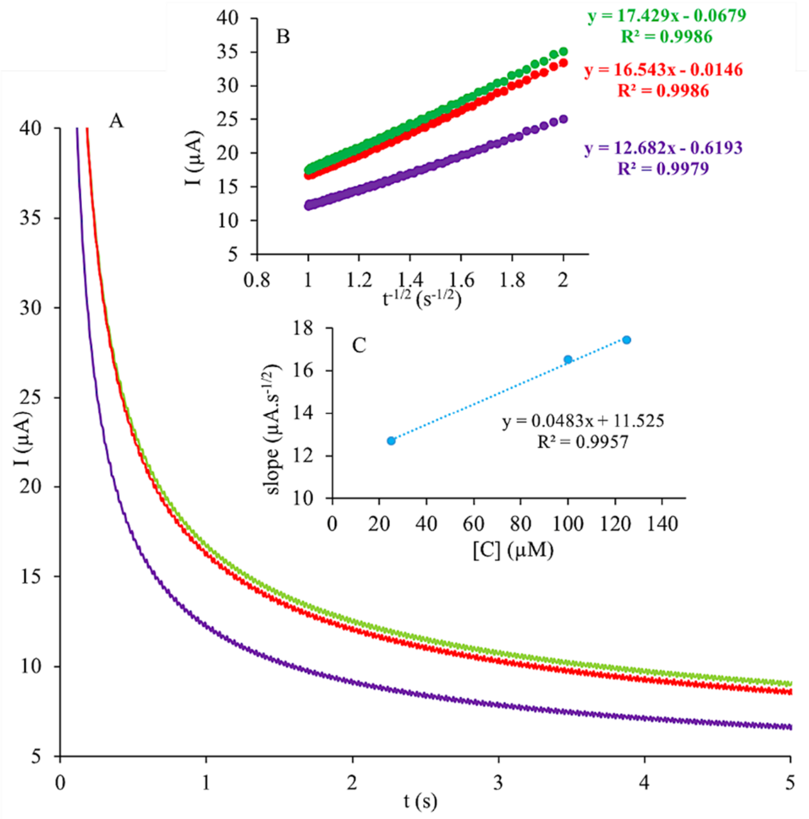

Figure S6. (A) Chronoamperograms of GCE/GO-MWCNT-CHT for varying concentrations of C: 25, 100, 125 µM in 0.2 M PBS (pH 7.0); (B) Plots of anodic peak currents (I_{pa}) vs. $t^{-1/2}$; (C) Plot of the slope of straight line vs. C concentration.

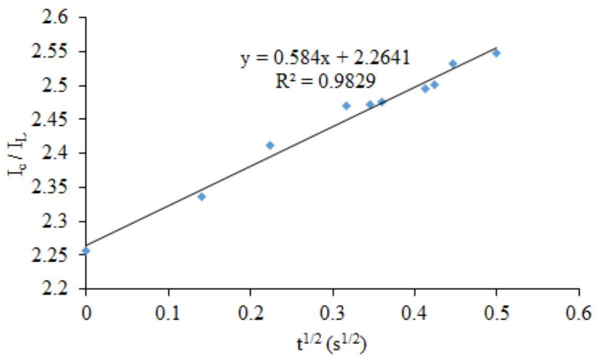

Figure S7. Linear dependence of square root of time on I_c/ I_L for UA detection using chronoamperometry.

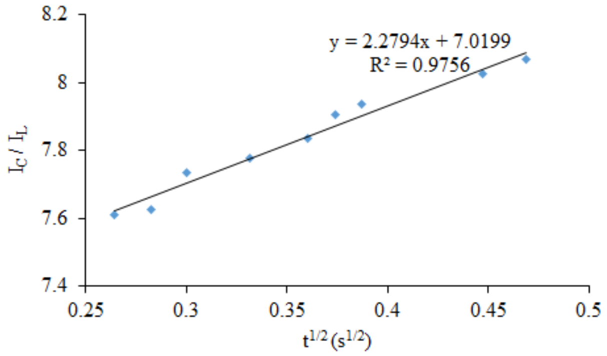

Figure S8. Linear dependence of square root of time on I_c/ I_L for G detection using chronoamperometry.

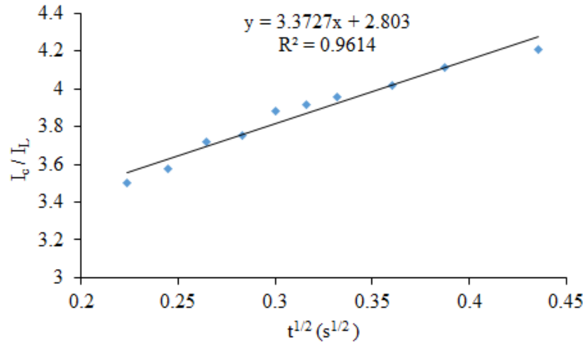

Figure S9. Linear dependence of square root of time on I_c/ I_L for A detection using chronoamperometry.

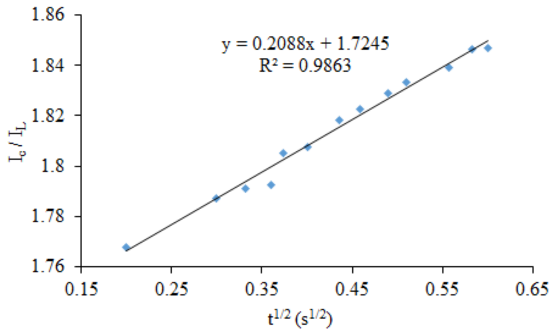

Figure S10. Linear dependence of square root of time on I_c/ I_L for T detection using chronoamperometry.

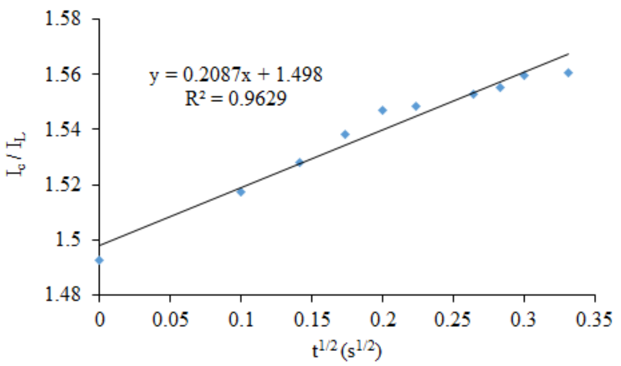

Figure S11. Linear dependence of square root of time on I_c/ I_L for C detection using chronoamperometry.

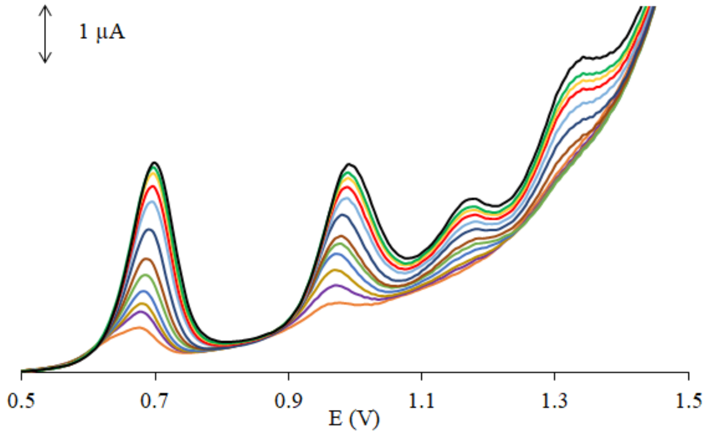

Figure S12. DPV for standard addition in human serum sample that was diluted 10-fold in 0.2 M PBS (pH 7.0).

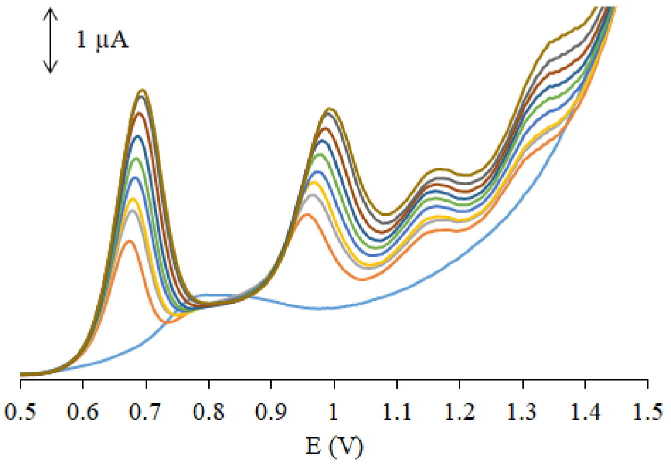

Figure S13. DPV for standard addition in human saliva sample that was diluted 5-fold in 0.2 M PBS (pH 7.0).

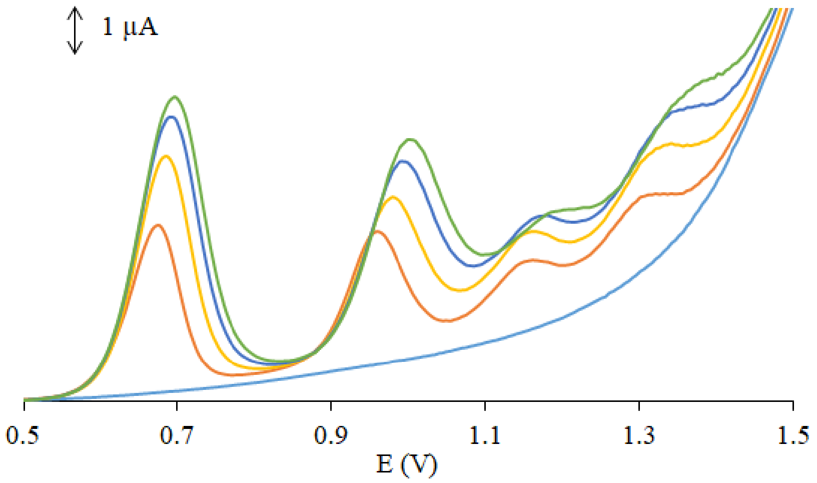

Figure S14. DPV for standard addition in artificial saliva sample that was diluted 5-fold in 0.2 M PBS (pH 7.0).
